# Supplementary material for: Who We Seek and What We Eat? Sources of Food Choice Inspirations and Their Associations with Adult Dietary Patterns before and during the COVID-19 Lockdown in New Zealand
Source: Nutrients. 2021 Nov 1;13(11):3917. doi: 10.3390/nu13113917 (PMC8617873; doi:10.3390/nu13113917)

Supplementary Files

Supplementary Table S1: Survey questions and response options for determining the variables of food and cooking-related advice sought and reasons for recipe selection.

**When you do grocery shopping, you make choices, and these choices can also be influenced by others (indirectly). How often did the following people/sources influence your usual food choices when you went grocery shopping? Before Lockdown and During Lockdown**	**Response Options**
Household members	Never, Very Rarely, Rarely, Sometimes, Frequently, Very Frequently, or Every time I prepared food
Family	Never, Very Rarely, Rarely, Sometimes, Frequently, Very Frequently, or Every time I prepared food
Friends/acquaintances	Never, Very Rarely, Rarely, Sometimes, Frequently, Very Frequently, or Every time I prepared food
Food advertisements/marketing	Never, Very Rarely, Rarely, Sometimes, Frequently, Very Frequently, or Every time I prepared food
Celebrity chefs	Never, Very Rarely, Rarely, Sometimes, Frequently, Very Frequently, or Every time I prepared food
Other celebrities	Never, Very Rarely, Rarely, Sometimes, Frequently, Very Frequently, or Every time I prepared food
Food influencers (not chefs either, people that generate food content via any medium to reach and engage with their followers)	Never, Very Rarely, Rarely, Sometimes, Frequently, Very Frequently, or Every time I prepared food
Other	Never, Very Rarely, Rarely, Sometimes, Frequently, Very Frequently, or Every time I prepared food
**Whose advice about the sorts of food you should eat for your health did you listen to? Before Lockdown and During Lockdown**	**Response Options**

Supplementary Files

Household members	Never, Very Rarely, Rarely, Sometimes, Frequently, Very Frequently, or Every time I needed or wanted advice
Family	Never, Very Rarely, Rarely, Sometimes, Frequently, Very Frequently, or Every time I needed or wanted advice
Friends and acquaintances	Never, Very Rarely, Rarely, Sometimes, Frequently, Very Frequently, or Every time I needed or wanted advice
Nutrition experts (dietitians or nutritionists)	Never, Very Rarely, Rarely, Sometimes, Frequently, Very Frequently, or Every time I needed or wanted advice
Health experts (medical doctors)	Never, Very Rarely, Rarely, Sometimes, Frequently, Very Frequently, or Every time I needed or wanted advice
Scientists	Never, Very Rarely, Rarely, Sometimes, Frequently, Very Frequently, or Every time I needed or wanted advice
Celebrity Chefs	Never, Very Rarely, Rarely, Sometimes, Frequently, Very Frequently, or Every time I needed or wanted advice
Food influencers (not chefs either, people that generate food content via any medium to reach and engage with their followers)	Never, Very Rarely, Rarely, Sometimes, Frequently, Very Frequently, or Every time I needed or wanted advice
Other people	Never, Very Rarely, Rarely, Sometimes, Frequently, Very Frequently, or Every time I needed or wanted advice
There are many recipes and food preparation messages available to us. How often did you actively search for recipes/food preparation content? Before Lockdown and During Lockdown	Never, Very Rarely, Rarely, Sometimes, Frequently, Very Frequently, or All the time
**When you select recipes, which of the following things do you take into consideration? Before Lockdown and During Lockdown**	**Response Options**

Supplementary Files

Guaranteed to taste good	Strongly disagree, disagree, somewhat disagree, neither agree nor disagree, somewhat agree, agree, or strongly agree
Achievable with few ingredients	Strongly disagree, disagree, somewhat disagree, neither agree nor disagree, somewhat agree, agree, or strongly agree
Achievable with the ingredients I have at home	Strongly disagree, disagree, somewhat disagree, neither agree nor disagree, somewhat agree, agree, or strongly agree
Achievable with ingredients that can be easily found at the store	Strongly disagree, disagree, somewhat disagree, neither agree nor disagree, somewhat agree, agree, or strongly agree
Easy to prepare	Strongly disagree, disagree, somewhat disagree, neither agree nor disagree, somewhat agree, agree, or strongly agree
Quick to prepare	Strongly disagree, disagree, somewhat disagree, neither agree nor disagree, somewhat agree, agree, or strongly agree
Innovative (new, something different)	Strongly disagree, disagree, somewhat disagree, neither agree nor disagree, somewhat agree, agree, or strongly agree
Inexpensive to make	Strongly disagree, disagree, somewhat disagree, neither agree nor disagree, somewhat agree, agree, or strongly agree
Comforting	Strongly disagree, disagree, somewhat disagree, neither agree nor disagree, somewhat agree, agree, or strongly agree
Healthy	Strongly disagree, disagree, somewhat disagree, neither agree nor disagree, somewhat agree, agree, or strongly agree
Environmentally friendly	Strongly disagree, disagree, somewhat disagree, neither

Supplementary Files

	agree nor disagree, somewhat agree, agree, or strongly agree
**Name your top food-related influential figure, organization or brand whose recipes you use the most. Before Lockdown and During Lockdown**	**Free text box**
**Now rate this person/organization/brand on the following criteria. Before Lockdown and During Lockdown**	**Response Options**
He/she/it was trustworthy	Strongly disagree, disagree, somewhat disagree, neither agree nor disagree, somewhat agree, agree, or strongly agree
He/she/it was an expert	Strongly disagree, disagree, somewhat disagree, neither agree nor disagree, somewhat agree, agree, or strongly agree
He/she/it was relatable	Strongly disagree, disagree, somewhat disagree, neither agree nor disagree, somewhat agree, agree, or strongly agree

Supplementary Files

CONSORT

TRANSPARENT REPORTING of TRIALS

Supplementary Figure S1: CONSORT 2010 Flow Diagram

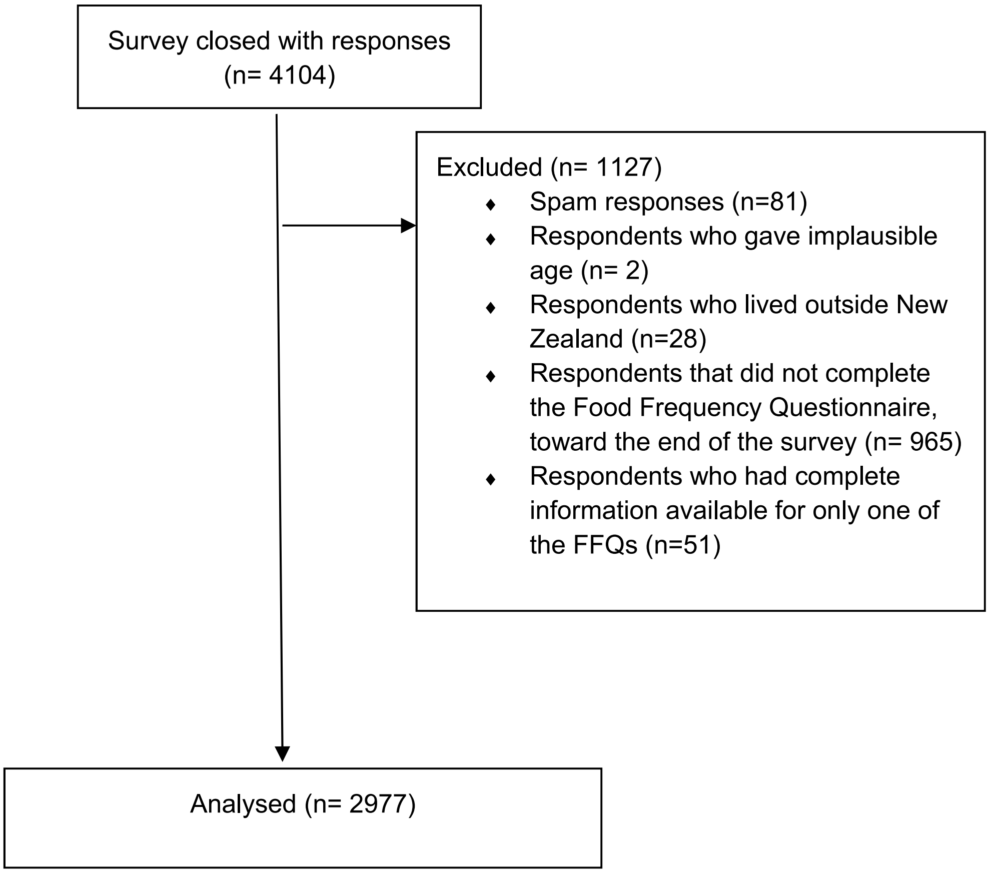

Supplement: Supplementary file 1 [file nutrients-13-03917-s001.zip › nutrients-1395669-supplementary.pdf]
